# How Often Do the Iranian Medical Journal Editors-in-Chief Publish in Their Own Journals?

**DOI:** 10.18502/ijph.v49i7.3592

**Published:** 2020-07

**Authors:** Mohammad Saeid REAEE-ZAVAREH, Hamidreza KARIMI-SARI

**Affiliations:** Middle East Liver Diseases (MELD) Center, Tehran, Iran

## Dear Editor-in-Chief

Although editors can certainly publish their papers in their own journals, evaluating these types of publications always need more caution (1). We know that a simple declaration of conflict of interest in this situation is not adequate at all and those editors should have no effect on the editorial decision about their submitted manuscript (2, 3). This situation needs a transparent review process that is solely independent of those editors (2). Therefore, managing high numbers of such papers in a journal can be difficult. Here, we tried to determine the proportion of published papers by Iranian medical editors in their own journals. In this project, we included all Iranian medical journals located in the Science Citation Index Expanded (SCIE). We extracted H-index and total published papers until the end of 2019 and during the last five years (2015 – 2019), for the chairperson, editor-in-chief (EIC), or director in charge of each included journal using Scopus database. Finally, we calculated the proportion of published papers by editors in his/her own journal in both of the aforementioned periods. According to the Scopus paper classification, we considered only two types of papers including articles and reviews, in all steps of our investigations.

**Fig. 1:**
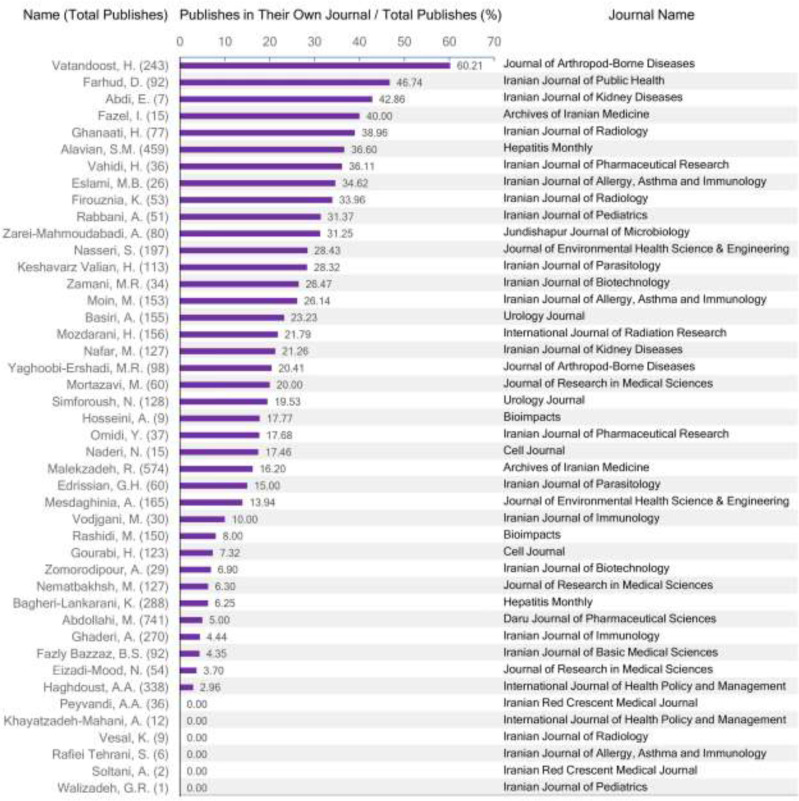
Proportion of published papers by the Iranian medical editors-in-chief, chairpersons, or directors in charge, in their own journals indexed in Science Citation Index Expanded

Table and Fig. 1 show the relationship between Iranian medical journals indexed in SCIE and their chairpersons, editors-in-chief, or directors in charge regarding the number of publications. The median (interquartile range) numbers of published papers by Iranian medical editors in their own journals are 17.72 (51.31 – 30.55) and 19.05 (2.63 – 36.33) until the end of 2019 and in 2015 – 2019 respectively. On average, Iranian editors have published 18.90% (standard deviation [SD] = 15.03) and 24.98% (SD = 27.45) of their papers in their own journals until the end of 2019 and in 2015 – 2019 respectively. There was a non-significant inverse relationship between the journals impact factor and the proportion of published papers by editors in their own journals until the end of 2019 (P = 0.36, Correlation coefficient [CC] = − 0.14) and in 2015–2019 (P = 0.31, CC = − 0.16).

One important point should be considered that none of the reported proportions in Table 1 can necessarily be considered as an unethical practice. Some editors may want to publish their best research projects in their own journals which is more common in very specialized journals (4). Similar to the self-citation rate for each author, we cannot determine an exact ethical ratio for the proportion of published papers by editors in their own journals, but journals should prepare a completely transparent peer-reviewing system for such manuscripts so that authors’ name omitted from those manuscripts during the review process (3). Additionally, in some cases, editors can publish a commentary paper about the review process of such manuscripts together with publishing them (5). We believe that preparing this system is very necessary for at least some of the Iranian journals. Furthermore, the proportions of published papers by editors in their journals can regularly be shared through the journal.

**Table 1:** Overview of the relationship between the Iranian medical journals indexed in science citation index expanded and their chairpersons, editors-in-chief, or directors in charge

*** Researcher Name (Role in the journal) ***	*** Journal Name (Impact Factor) ***	*** H-Index ***	*** Total Papers ***		*** Editor’s Papers in Journal / Editor’s Papers (%) ***	
			*** All Periods ***	*** 2015 – 2019 ***	*** All Periods ***	*** 2015–2019 ***
Vatandoost, Hassan (Editor-in-Chief)	Journal of Arthropod-Borne Diseases (1.231)	34	243	93	60.21	40.86
Farhud, Dariush (Chairman & Editor-in- Chief)	Iranian Journal of Public Health (1.225)	15	92	16	46.74	81.25
Abdi, Ezatollah (Chairman)	Iranian Journal of Kidney Diseases (1.200)	4	7	1	42.86	100.00
Fazel, Iradj (Chairman)	Archives of Iranian Medicine (1.200)	8	15	1	40.00	0.00
Ghanaati, Hossein (Chairman)	Iranian Journal of Radiology (0.478)	14	77	17	38.96	70.59
Alavian, Seyed Moayed (Editor-in-Chief)	Hepatitis Monthly (1.578)	41	459	177	36.60	39.55
Vahidi, Hossein (Director-in-Charge)	Iranian Journal of Pharmaceutical Research (1.183)	9	36	21	36.11	47.62
Eslami, Mohammad Bagher (Editor-in-Chief)	Iranian Journal of Allergy, Asthma and Immunology (1.222)	10	26	1	34.62	100.00
Firouznia, Kavous (Editor-in-Chief)	Iranian Journal of Radiology (0.478)	10	53	12	33.96	66.67
Rabbani, Ali (President)	Iranian Journal of Pediatrics (0.587)	11	51	17	31.37	35.29
Zarei-Mahmoudabadi, Ali (Editor-in-Chief)	Jundishapur Journal of Microbiology (0.957)	15	80	27	31.25	37.04
Nasseri, Simin (Editor-in-Chief)	Journal of Environmental Health Science & Engineering (2.773)	31	197	86	28.43	19.77
Keshavarz Valian, Hosein (Chairman)	Iranian Journal of Parasitology (0.735)	17	113	51	28.32	27.45
Zamani, Mohammad Reza (Chairman)	Iranian Journal of Biotechnology (0.861)	8	34	16	26.47	18.75
Moin, Mostafa (Chairman)	Iranian Journal of Allergy, Asthma and Immunology (1.222)	30	153	31	26.14	32.26
Basiri, Abbas (Editor-in-Chief)	Urology Journal (1.463)	26	155	39	23.23	20.51
Mozdarani, Hossein (Editor-in-Chief)	International Journal of Radiation Research (0.514)	21	156	48	21.79	31.25
Nafar, Mohsen (Editor-in-Chief)	Iranian Journal of Kidney Diseases (1.200)	19	127	53	21.26	20.75
Yaghoobi-Ershadi, Mohammad Reza (Chairman)	Journal of Arthropod-Borne Diseases (1.231)	27	98	30	20.41	36.67
Mortazavi, Mojgan (Editor-in-Chief)	Journal of Research in Medical Sciences (1.467)	14	60	22	20.00	13.64
Simforoush, Nasser (Director)	Urology Journal (1.463)	22	128	22	19.53	27.27
Omidi, Yadollah (Editor-in-Chief)	Bioimpacts (3.190)	38	198	100	17.68	20.00
Naderi, Nima (Editor-in-Chief)	Iranian Journal of Pharmaceutical Research (1.183)	16	63	12	17.46	33.33
Hosseini, Ahmad (Editor-in-Chief)	Cell Journal (2.046)	9	45	31	17.77	19.35
Malekzadeh, Reza (Editor-in-Chief)	Archives of Iranian Medicine (1.200)	88	574	257	16.20	14.01
Edrissian, Gholam Hossein (Editor-in-Chief)	Iranian Journal of Parasitology (0.735)	19	60	5	15.00	80.00
Mesdaghinia, Alireza (Chairman)	Journal of Environmental Health Science & Engineering (2.773)	27	165	60	13.94	13.33
Vodjgani, Mohammad (Chairman)	Iranian Journal of Immunology (0.937)	11	30	6	10.00	0.00
Rashidi, Mohammad-Reza (Director-in-Charge)	Bioimpacts (3.190)	26	150	55	8.00	7.27
Gourabi, Hamid (Chairman)	Cell Journal (2.046)	27	123	45	7.32	8.89
Zomorodipour, Alireza (Editor-in-Chief)	Iranian Journal of Biotechnology (0.861)	12	29	7	6.90	14.29
Nematbakhsh, Mehdi (Chairman)	Journal of Research in Medical Sciences (1.467)	24	127	49	6.30	2.04
Bagheri-Lankarani, Kamran (Chairman)	Hepatitis Monthly (1.578)	28	288	154	6.25	3.25
Abdollahi, Mohammad (Editor-in-Chief)	Daru Journal of Pharmaceutical Sciences (2.698)	72	741	237	5.00	2.53
Ghaderi, Abbas (Editor-in-Chief)	Iranian Journal of Immunology (0.937)	32	270	81	4.44	1.23
Fazly Bazzaz, Bibi Sedigheh (Editor-in-Chief)	Iranian Journal of Basic Medical Sciences (1.854)	22	92	46	4.35	4.35
Eizadi-Mood, Nastaran (EMERITA EIC)	Journal of Research in Medical Sciences (1.467)	11	54	20	3.7	5.00
Haghdoust, Ali Akbar (Director-in-Charge)	International Journal of Health Policy and Management (4.485)	30	338	170	2.96	2.94
Peyvandi, Ali Asghar (Chairman)	Iranian Red Crescent Medical Journal (0.644)	10	36	17	0.00	0.00
Khayatzadeh-Mahani, Akram (Editor-in-Chief)	International Journal of Health Policy and Management (4.485)	5	12	8	0.00	0.00
Vessal, Karim (Editor-in-Chief)	Iranian Journal of Radiology (0.478)	6	9	1	0.00	0.00
Rafiei Tehrani, Shahnaz (Editor-in-Chief)	Iranian Journal of Allergy, Asthma and Immunology (122)	4	6	0	0.00	0.00
Soltani, Ahmad (Editor-in-Chief)	Iranian Red Crescent Medical Journal (0.644)	1	2	2	0.00	0.00
Walizadeh, Gholam Reza (Editor-in-Chief)	Iranian Journal of Pediatrics (0.587)	1	1	0	0.00	0.00

Our project was a preliminary study and we did not include associate editors and did not consider the period that a researcher had been a journal editor. For future projects, we suggest calculations of these proportions among different countries and in various fields. This provides us the possibility of inter-fields and -countries comparisons for getting better perspectives.
